# DNA Methylation Combinations in Adjacent Normal Colon Tissue Predict Cancer Recurrence: Evidence from a Clinical Cohort Study

**DOI:** 10.1371/journal.pone.0123396

**Published:** 2015-03-27

**Authors:** Jen Chun Kuan, Chang Chieh Wu, Chien An Sun, Chi Ming Chu, Fu Gong Lin, Chih Hsiung Hsu, Po-Chieh Kan, Shih-Chieh Lin, Tsan Yang, Yu-Ching Chou

**Affiliations:** 1 Graduate Institute of Life Sciences, National Defense Medical Center, Taipei, Taiwan (R.O.C.); 2 Department of Surgery, Tri-Service General Hospital, National Defense Medical Center, Taipei, Taiwan (R.O.C.); 3 Department of Public Health, College of Medicine, Fu-Jen Catholic University, New Taipei City, Taiwan (R.O.C.); 4 School of Public Health, National Defense Medical Center, Taipei, Taiwan (R.O.C.); 5 Graduate Institute of Medical Sciences, National Defense Medical Center, Taipei, Taiwan (R.O.C.); 6 Department of Health Business Administration, Meiho University, Pingtung, Taiwan; Sapporo Medical University, JAPAN

## Abstract

Accumulating evidence has suggested the requirement for further stratification of patients in the same tumor stage according to molecular factors. We evaluate the combination of cancer stage and DNA methylation status as an indicator of the risk of recurrence and mortality among patients with colorectal cancer (CRC). A cohort study of 215 patients with CRC (mean age 64.32 years; 50.5% of men) from Tri-Service General Hospital in Taiwan examined the association between cancer stage and risk of CRC recurrence and mortality. A Cox proportional hazard model was used to analyze patient methylation status and clinical information at study entry, and their associations with CRC recurrence and mortality during follow-up. The advanced stage patients with *p16*, *hMLH1*, and *MGMT* methylation were associated with higher risk of CRC recurrence compared with the local stage patients with unmethylation status in tumor tissues, with adjusted hazard ratios (HRs) (95% confidence interval [CI]) of 9.64 (2.92–31.81), 8.29 (3.40–20.22), and 11.83 (3.49–40.12), respectively. When analyzing normal tissues, we observed similar risk of CRC recurrence with adjusted HRs (95% CI) of 10.85 (4.06–28.96), 9.04 (3.79–21.54), and 12.61 (4.90–32.44), respectively. For combined analyses, the risk of recurrence in the patients in advanced stage with DNA methylation in both normal and tumor tissues, compared with local stage with unmethylation, was increased with adjusted HR (95% CI) of 9.37 (3.36–26.09). In the advanced stage patients, methylation status and tissue subtype were associated with increased risk of 5-year cumulative CRC recurrence (*p* < 0.001). This study demonstrates that clustering DNA methylation status according to cancer stage and tissue subtype is critical for the assessment of risk of recurrence in CRC patients and also indicated an underlying mechanism.

## Introduction

Colorectal cancer (CRC) is one of the most common cancers, with over 140000 new cases and 50,830 deaths estimated to have occurred in the United States in 2013 [1]. Although the incidence and mortality rates of CRC have decreased considerably in recent years because of substantial improvements in screening and treatment strategies, the prognosis of CRC patients after surgical resection remains poor [2].

The current guidelines for the assessment of the prognosis of CRC patients emphasize the classification of individual tumor-node-metastasis elements [3]. However, accumulating evidence has suggested the requirement for further stratification of patients in the same tumor stage according to molecular factors [4]. Furthermore, the molecular factors, which include biomarkers of several cancers, could have potential clinical value in predicting cancer development and progression, and predicting the prognosis of cancer patients [5,6].

CRC is recognized as a consequence of the accumulation of genetic (gene mutations) and epigenetic alterations (aberrant DNA methylation) that transform colonic epithelial cells into colon adenocarcinoma cells [7]. Epigenetic modification, which involves DNA and histone modifications, plays a critical role in carcinogenesis pathways in several cancers. Previous studies have demonstrated that the most frequent aberrant epigenetic modification in cancer, DNA methylation, reduces the effectiveness of the RNA transcription of tumor suppressor genes [8,9]. Recent study has concluded that several gene promoter methylations result in transcriptional silencing, and could be exploited as biomarkers for the early detection of CRC [10]. Tumor progression in CRC is caused by loss of function in cell-cycle regulation and DNA mismatched repair (MMR), which is related to the DNA methylation of the transcription start site [11]. Previous studies have identified the DNA-methylation-related genes associated with carcinogenesis pathways using gene silencing [12,13]. However, these studies did not analyze DNA methylation status and clinical stage for the follow-up of patients with CRC.

The purpose of this study was to evaluate the combination of cancer stage and DNA methylation status as an indicator of the risk of recurrence and mortality among patients with CRC. We also investigated the association between the risk factors for and prognosis of CRC, after stratifying according to colon tissue subtypes.

## Materials and Methods

### Patient characteristics

According to the written operating procedures, Good Clinical Practice (GCP), and the applicable regulatory requirements, this application was approved by Tri-Service General Hospital Institutional Review Board (IRB) (TSGHIRB approval number: 098-05-292). The board was organized under, and operates per International Conference on Harmonization (ICH) / WHO GCP and the applicable laws and regulations. All patients signed informed consent letter and were clinically diagnosed with CRC had to be able to tolerate surgical resection. Patient characteristics (sex, age at surgery, stage, recurrence, and mortality) were obtained from their medical histories. Pairs of specimens were collected from the 215 CRC patients (430 samples) who fulfilled the inclusion criteria. Sample collection was performed at surgical clinics, in which tumor and normal tissues were resected simultaneously. The adjacent normal tissue specimens were collected from an incision > 10 cm away from the carcinoma sites. All specimens were immediately stored in liquid nitrogen. The resection procedure was reviewed by the Department of Colorectal Surgery, Tri-Service General Hospital.

### DNA isolation and sodium bisulfite treatment

Genomic DNA was extracted from tissue samples by using a MagCore Automated Nucleic Acid Extractor (RBC Bioscience, Taipei, Taiwan) and a Genomic DNA Tissue Kit according to the manufacturer’s protocol. The resulting DNA was sodium bisulfite-modified using an EZ DNA Methylation Kit (Zymo Research, Orange, USA). A methylated DNA control for methylation-specific polymerase chain reaction (MS-PCR) assays was generated using SssI methylase (Zymo Research, Orange, USA).

### Methylation-specific PCR assay

Selected 3 tumor suppressor genes (*p16*, *hMLH1*, and *MGMT*) are related to risk of CRC which involved in the pathways associated with cancer stages and prognosis, including MMR and the cell cycle [14]. Conditions of DAN bisulfite-treatment and methylation-specific PCR assay were provided in **S1 Methods**, and quality control of the assay was provide in **S1 Fig**.

### Statistical analysis

Patient baseline characteristics, including sex, age at surgery (continuous), stage (I, II, III, and IV), recurrence, mortality status, and gene promoter region methylation status in the candidate genes (*p16*, *hMLH1*, and *MGMT*), were compared. To evaluate the possible interactions between DNA methylation and clinical stage, and risk of CRC recurrence and mortality, patients of different cancer stages were evaluated separately and also divided into 2 subgroups (local and advanced). Tumor tissues were compared with adjacent normal tissues. Several factors could have contributed to the differences between our results and those of other studies. Confounding factors adjustment is detailed in **S1 Methods**. All statistical analyses were performed using SPSS software (IBM SPSS Statistics 21; Asia Analytics Taiwan Ltd., Taipei, Taiwan). All statistical tests were 2-sided with an α level of 0.05.

## Results

The mean age (± SD) of the study patients was 64.32 (± 14.48) years, and men constituted 50.5% of the study sample. Overall, the mean duration (± SD) of follow-up was 24.94 (± 17.90) months. During the 5,363 person-months of follow-up, we identified 85 cases of CRC recurrence and 43 deaths, with an incidence density of 15.85 and 8.02 per 1000 person-months, respectively. The association between clinical stage and risk of CRC recurrence and mortality is provide in **S1 Results**.

**Fig 1:**
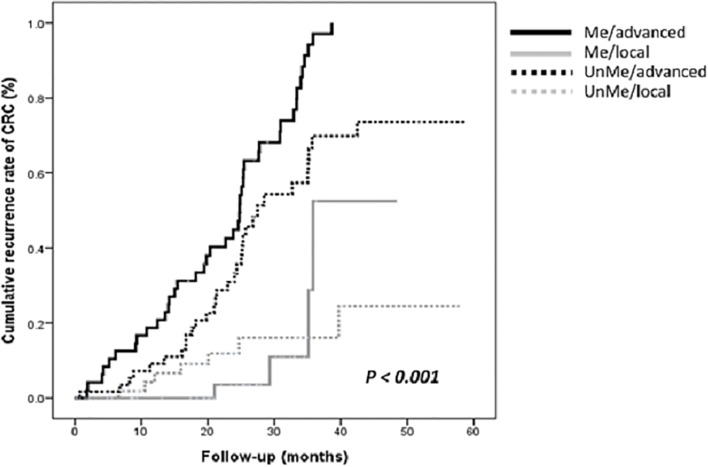
Kaplan-Meier estimates of cumulative recurrence rate of CRC was stratified into 4 subgroups: unmethylated in local stage (UnMe/local), unmethylated in advanced stage (UnMe/advanced), methylated in local stage (Me/local), and methylated in advanced stage (Me/advanced), respectively. The cumulative recurrence rates were significantly different in the 4 subgroups during the 5-years of follow-up after adjusted for sex, age at surgery, tumor location, adjuvant chemotherapy, and clinical stage.

Table 1 displays the baseline characteristics of the patients according to their DNA methylation status. When analyzing normal tissues, we observed that the patients with higher methylation status of the tumor suppressor genes were significantly associated with a higher proportion of CRC recurrence than patients with lower methylation status were (55.3% vs. 37.7%, *p* = 0.012). This association was borderline significant in tumor tissues (84.7% vs. 73.1%, *p* = 0.065). We further examined the association between cancer stage and risk of CRC recurrence, stratifying by target gene methylation status in the tissue subtypes after adjusted for gender, age at surgery, and adjuvant chemotherapy (Table 2). When analyzing tumor tissues, we observed that the advanced stage patients with *p16*, *hMLH1*, and *MGMT* methylation were associated with higher risk of CRC recurrence compared with the local stage patients with unmethylation status, with adjusted HRs (95% CI) of 9.64 (2.92–31.81), 8.29 (3.40–20.22), and 11.83 (3.49–40.12), respectively. When analyzing normal tissues, we observed similar risk of CRC recurrence in the advanced stage patients with *p16*, *hMLH1*, and *MGMT* methylation compared with the local stage patients with unmethylation status, with adjusted HRs (95% CI) of 10.85 (4.06–28.96), 9.04 (3.79–21.54), and 12.61 (4.90–32.44), respectively. Furthermore, a Kaplan-Meier analysis with log-rank test showed the significance of the association between cancer stage and DNA methylation status in adjacent normal tissues, and the 5-year cumulative recurrence of CRC (*p* < 0.001; Fig. 1); a Cox regression model confirmed the consistent risk after adjusted potential confounding factors, such as sex, age, tumor location, adjuvant chemotherapy, and clinical stage (*p* < 0.001).

**Table 1:** Baseline characteristics of subjects according to the distribution of DNA methylation in subtypes of tissues.

Variables	***Total***	Methylation status							
		***p16***		***MLH1***		***MGMT***		≥ 1 of genes	
		Normal	Tumor	Normal	Tumor	Normal	Tumor	Normal	Tumor
Sex, n (%)									
Male	109 (50.5)	27 (25.7)	66 (60.6)	19 (17.4)	24 (22.0)	18 (16.5)	63 (57.8)	45 (41.3)	87 (79.8)
Female	106 (49.5)	35 (33.0)	52 (49.1)	19 (17.9)	25 (23.6)	13 (12.3)	56 (52.8)	51 (48.1)	80 (75.5)
*P* value ^a^		0.294	0.101	0.924	0.871	0.439	0.495	0.339	0.513
Age at surgery									
Mean (SD)	64.3 (14.5)	62.7 (14.3)	63.2 (15.7)	66.2 (12.3)	67.4 (12.9)	61.5 (15.2)	63.9 (14.3)	63.3 (14.6)	64.0 (15.0)
≤ 65, n (%)	107 (49.8)	32 (29.9)	63 (58.9)	18 (16.8)	21 (19.6)	19 (17.8)	62 (57.9)	51 (47.7)	87 (81.3)
> 65, n (%)	108 (50.2)	31 (28.7)	55 (50.9)	20 (18.5)	28 (25.9)	12 (11.1)	57 (52.8)	45 (41.7)	80 (74.1)
*P* value ^a^		0.882	0.274	0.858	0.330	0.179	0.494	0.412	0.252
Stage, n (%)									
I	29 (13.6)	10 (34.5)	12 (41.4)	3 (10.3)	6 (20.7)	2 (6.9)	12 (41.4)	12 (41.4)	19 (65.5)
II	78 (36.4)	23 (30.4)	45 (57.0)	13 (16.5)	16 (20.3)	9 (11.4)	44 (55.7)	35 (44.3)	63 (79.7)
III	71 (33.2)	20 (28.2)	39 (54.9)	17 (23.9)	18 (25.4)	11 (15.5)	44 (62.0)	33 (46.5)	58 (81.7)
IV	36 (16.8)	9 (25.0)	22 (61.1)	5 (13.9)	9 (25.0)	9 (25.0)	19 (52.8)	16 (44.4)	27 (75.0)
*P* value ^a^		0.853	0.417	0.331	0.867	0.154	0.302	0.973	0.324
Recurrence, n (%)									
Absence	130 (60.7)	32 (24.6)	64 (49.2)	19 (14.6)	27 (20.8)	13 (10.0)	68 (52.3)	49 (37.7)	95 (73.1)
Presence	84 (39.3)	30 (36.5)	54 (63.5)	19 (22.4)	22 (25.9)	18 (21.2)	51 (60.0)	47 (55.3)	72 (84.7)
*P* value ^a^		0.068	0.027	0.200	0.409	0.029	0.326	0.012	0.065
Mortality, n (%)									
Absence	172 (79.9)	50 (29.1)	97 (56.4)	32 (18.6)	41 (23.8)	24 (14.0)	98 (57.0)	79 (45.9)	137 (79.7)
Presence	43 (20.1)	13 (30.2)	21 (48.8)	6 (14.0)	8 (18.6)	7 (16.3)	21 (48.8)	17 (39.5)	30 (69.8)
*P* value ^a^		0.854	0.236	0.655	0.546	0.808	0.392	0.496	0.218

^a^ *P* value for between group comparison with regard to methylation status in normal and tumor tissues.

**Table 2:** Risk of CRC recurrence during follow-up in relation to methylation status in patients with colorectal cancer after surgery.

Recurrence in different stages by methylation	Normal tissues							Tumor tissues						
	No. of subjects		Incidence rate	Crude		Adjusted		No. of subjects		Incidence rate	Crude		Adjusted	
				HR	95% CI	HR	95% CI				HR	95% CI	HR	95% CI
***p16***														
UnMe/local (1&2) ^a^	74	(1767)	4.53	1.00	(Referent)	1.00	(Referent)	51	(1358)	4.42	1.00	(Referent)	1.00	(Referent)
UnMe/advanced (3&4) ^b^	78	(1941)	23.70	5.08	(2.40–10.77)	6.07	(2.35–15.70)	46	(1180)	21.19	4.69	(1.89–11.65)	6.63	(1.97–22.38)
Me/local (1&2)^c^	34	(1032)	3.88	0.70	(0.18–2.62)	0.98	(0.23–4.17)	57	(1442)	4.16	0.87	(0.28–2.70)	1.15	(0.27–4.94)
Me/advanced (3&4) ^d^	29	(621)	43.48	9.92	(4.47–21.99)	10.85	(4.06–28.96)	61	(1383)	34.71	8.01	(3.34–19.16)	9.64	(2.92–31.81)
***MLH1***														
UnMe/local (1&2)^a^	92	(2411)	4.98	1.00	(Referent)	1.00	(Referent)	86	(2148)	5.12	1.00	(Referent)	1.00	(Referent)
UnMe/advanced (3&4) ^b^	85	(2098)	25.74	5.28	(2.76–10.11)	5.79	(2.67–12.57)	80	(1960)	26.53	5.37	(2.73–10.57)	6.08	(2.66–13.89)
Me/local (1&2) ^c^	16	(389)	NA^e^	NA^e^	NA^e^	NA^e^	NA^e^	22	(652)	1.53	0.30	(0.04–2.32)	0.48	(0.06–3.90)
Me/advanced (3&4) ^d^	22	(465)	40.86	8.71	(4.12–18.41)	9.04	(3.79–21.54)	27	(603)	34.83	6.67	(3.14–14.17)	8.29	(3.40–20.22)
***MGMT***														
UnMe/local (1&2) ^a^	97	(2625)	4.19	1.00	(Referent)	1.00	(Referent)	52	(1424)	3.51	1.00	(Referent)	1.00	(Referent)
UnMe/advanced (3&4) ^b^	87	(2154)	26.00	6.50	(3.32–12.74)	7.63	(3.34–17.47)	44	(1166)	24.87	6.61	(2.52–17.37)	7.61	(2.23–25.91)
Me/local (1&2)^c^	11	(176)	5.68	1.93	(0.24–15.17)	3.04	(0.37–25.05)	56	(1376)	5.09	1.32	(0.42–4.15)	1.48	(0.35–6.25)
Me/advanced (3&4) ^d^	20	(408)	41.67	11.71	(5.32–25.76)	12.61	(4.90–32.44)	63	(1396)	31.52	9.41	(3.66–24.17)	11.83	(3.49–40.12)

HR, hazard ratio; CI, confidence interval; N/A, not available.

Adjusted for gender, age at surgery (continuous), and adjuvant chemotherapy.

^a^ UnMe/loccal (1&2): gene promoter region unmethylation in cancer stage 1 or 2.

^b^ UnMe/advanced (3&4): DNA promoter region unmethylation in cancer stage 3 or 4.

^c^ Me/local (1&2): DNA promoter region methylation in cancer stage 1 or 2.

^d^ Me/advanced (3&4): DNA promoter region methylation in cancer stage 3 or 4.

^e^ Not available due to limited numbers of cases.

As shown in Table 3, we subsequently analyzed DNA methylation status and CRC recurrence in the study patients, stratifying according to cancer stage (local and advanced) and tissue subtype (normal and tumor). We observed that the risk of CRC recurrence was significantly associated with methylation status in the patients of the various cancer stages, and in the 2 tissue subtypes, after adjusting for sex and age at surgery (*p* < 0.001). The local stage patients were nonsignificantly associated with increased risk of CRC recurrence, irrespective of methylation status and tissue subtype. In the advanced stage patients, methylation status and tissue subtype were associated with increased risk of CRC recurrence.

**Table 3:** Risk of CRC recurrence in cluster of genes methylation status according to cancer stages and subtype of tissues.

Recurrence in different stages by methylation			Combined ≥ 1 of genes ^a^						
			No. of subjects		Incidence rate	Crude		Adjusted	
						HR	95% CI	HR	95% CI
Local	N & T	U	48	(1174)	3.41	1.00	(Referent)	1.00	(Referent)
Local	T	M	65	(1768)	4.52	1.15	(0.34–3.88)	1.06	(0.31–3.56)
Local	N	M	4	(216)	4.62	1.20	(0.12–11.73)	1.16	(0.12–11.30)
Local	N & T	M	99	(2442)	4.91	1.20	(0.39–3.72)	1.13	(0.36–3.51)
Advanced	N & T	U	42	(1271)	15.74	4.04	(1.36–11.97)	3.49	(1.17–10.42)
Advanced	T	M	60	(1403)	28.50	7.18	(2.53–20.37)	6.60	(2.32–18.77)
Advanced	N	M	2	(47)	42.90	11.25	(2.04–62.14)	11.49	(2.06–63.89)
Advanced	N & T	M	110	(2404)	34.94	9.93	(3.58–27.52)	9.37	(3.36–26.09)
*P* for trend						<0.001		<0.001	

HR, hazard ratio; CI, confidence interval; N, normal tissues; T, tumor tissues; U, unmethylation; M, methylation.

Adjusted for gender, age at surgery (continuous).

^a^ Combined ≥ 1 of genes had clustered *p16*, *MLH1* and *MGMT* promoter methylation status.

## Discussion

Our results indicated that the DNA methylation of certain genes and clinical stage were associated with the risk of CRC recurrence in our study patients. We observed that DNA methylation status in the 3 target genes and cancer stage were significantly associated with CRC recurrence in tumor and normal tissues. When we clustered the gene methylation statuses according to cancer stage and tissue subtype, we observed further increases in the risk of CRC recurrence. These results suggested that clinical cancer stage and DNA methylation status could potentially be evaluated as predictors of the risk of CRC recurrence.

Previous studies similarly observed an association between short recurrence-free survival and DNA methylation in CRC patients [15–17]. Zitt et al compared various screening tests for CRC, and reported that DNA methylation provides a clinically useful indicator for the early detection of disease progression in patients with CRC, which could potentially improve patient survival and quality of life [15]. Kim et al reported that the DNA methylation of multiple promoters could provide a biomarker for the early detection of CRC, and that DNA methylation patterns could also be used as predictors of metastatic or aggressive CRC [16]. The data from a clinical follow-up study revealed that the methylation of specific genes is associated with poor survival in advanced CRC patients [17]. A large follow-up study on 61 494 Asian and Caucasian patients with CRC indicated that the rates of survival were significantly higher in the patients of Filipino and Chinese ethnicity than in other patients [18]. Nevertheless, our results from a Chinese population were consistent with those of the described studies and the study had similar results with The Cancer Genome Atlas [19].

Aberrations in the methylation of macromolecules, particularly DNA, as well as disruption in DNA synthesis and repair, are considered to play major roles in carcinogenesis [20]. DNA methylation is the most extensively investigated epigenetic change [21], and can provide a new generation of cancers. Epigenetic modifications, particularly DNA methylation in selected gene promoters, are common molecular-level alterations in human tumors [16]. In this study, we observed that the methylation of certain genes was associated with significantly increased risk of CRC recurrence. Brock et al identified the methylation of *p16* and *CDH13* in tumorous and mediastinal lymph nodes, estimating the odds ratios (ORs) for CRC recurrence as 8.00 (95% CI, 2.50–25.51) and 4.32 (95% CI, 1.61–185.02), respectively. When they evaluated overall methylation in the tumorous and mediastinal lymph nodes, the OR for CRC recurrence was 15.5 (1.61–185.02) [22]. Our results similarly demonstrated increased risk of CRC recurrence when stratifying DNA methylation status according to clinical cancer stage and tissue subtype.

The genes evaluated in this study, *p16*, *hMLH1*, and *MGMT*, are involved in cell-cycle regulation and DNA MMR. Aberrant promoter methylation of the tumor suppressor genes can lead to downregulated transcriptional expression and protein expression in epithelial cells [23]. MMR and cell-cycle control both play crucial roles in the deletion of mononucleotide repeats, and loss of the repair system is one of the principal mechanisms involved in the accumulation of functional-change oncogenic effects, including CRC development [24]. DNA methylation alterations account for the histological heterogeneity and clinicopathological diversity of human cancers. Overexpression of DNA methyltransferase 1 is not a secondary result of increased cell proliferative activity, but is significantly correlated with accumulated DNA hypermethylation in the CpG islands of tumor-related genes [25]. The association between DNA methylation and cancer stage might be dependent on interleukin 6, a chronic inflammation marker that enhances the hypermethylation of tumor suppressor gene *p53* families, but reduces the methylation of the epidermal growth factor receptor [26]. Chronic inflammation is considered to be caused by inflammation-mediated cytosine damage, and the products of such damage might provide a mechanistic link between inflammation and cancer [27], and promote aberrant methylation in human cancers. Inflammatory response mediators, such as cytokines, free radicals, and growth factors, induce epigenetic changes in tumor suppressor genes. Observations of such changes have further strengthened the association between inflammation and cancer [28].

Therefore, the risk of poor prognosis increases in the advanced clinical stages of cancer when patients are first diagnosed, even after surgical resection. Our results were consistent with those of related studies associating lymphatic and distant metastases with poor prognosis [29,30]. The advanced stages of CRC are associated with higher risk of poor prognosis compared with local stage CRC. In addition, a clinical review of lung cancer indicated that when strategies are in place to expedite the examination of people suspected of having cancer, the disease typically shifts toward an earlier stage and resection rates increase [31]. Previous studies have identified that aberrant promoter hypermethylation of cancer-related genes may be potentially useful as epigenetic markers for predicting outcome of cancer treatment [32,33]. In the other study from 22 non-small cell lung cancer (NSCLC) patients by Esteller et al., 68% of the patients showing hypermethylation in tumor DNA also demonstrated in serum DNA across stages, which occurred with all stage from I to IIIA [33]. Two studies showed that aberrant DNA methylation are associated with cancer prognosis, even in different clinical stages. Another study showed the gene promoter methylation is associated with early recurrence of stage I NSCLC [22]. Additionally, the proportions of methylation were mostly lower in normal tissues than in tumor tissues in Table 1, but the risks of recurrence were similar in these two types of tissues. The results indicate that once the DNA methylation was detected in advanced patients, the risk of recurrence would be increased sharply. Previous study has showed the mechanism of this increased risk from the changes of molecular level. Sato et al. have reported DNA methylation of non-cancerous tissues obtained from patients with lung adenocarcinomas was at precancerous stages with aberrant DNA methylation over several genes [34]. DNA methylation alterations at precancerous stages are strengthened in normal tissues during the progression to developed lung adenocarcinoma. Which is consisted with our findings that the molecular changes might not be found by pathological examination, but the DNA methylation alterations have occurred in adjacent normal tissues from the lesions. Therefore, the DNA methylation alterations in normal tissues lead to a poor prognosis than in cancer tissues. Gene methylation status in an independent validation cohort was conducted with significantly higher odds ratio of recurrence among the case patients, typically increased with different sites of methylated detection, such as tumor, regional nodes, mediastinal nodes, and tumor and mediastinal nodes. Further investigations of patient clinical information, such as blood biochemical data or medication history, with stratification to identify high risk groups of CRC recurrence, are required.

The strengths and limitations of our study warrant discussion. In this study, we evaluated follow-up data on DNA methylation and cancer stage as indicators of risk of CRC recurrence according to tissue subtype. We performed our evaluations by using molecular biomarkers and clinical information. Our results could provide physicians with a clinical reference for the prognosis of CRC patients postsurgery. However, it is conceivable that longer time of follow-up may have greater differences in this study. Nevertheless, previous studies showed the short median of follow-up after surgical treatment among the CRC patients, under 2 years of follow-up periods, which are reported significant differences in incidence of recurrence [35,36]. Secondly, we did not evaluate inflammatory responses or inflammation-related biomarkers in the CRC patients. Kanai et al. identified that chronic inflammation can affect the prognosis of CRC patients [25]. We were unable to evaluate this association. Although, semi-quantitative MSP was used in this study, quantitative MPS such as, pyro-sequencing should be performed for further study. Moreover, gene expression did not assess in this study due to the frozen tissues stored in the tumor bank, the total RNA level in the specimen were insufficient for experiment of gene expression. Replication was not performed in this study.

In conclusion, this study demonstrates that clustering DNA methylation status according to cancer stage and tissue subtype is critical for the assessment of risk of recurrence in CRC patients. The study results also indicated an underlying mechanism for increased risk of recurrence in CRC patients, particularly in nontumorous tissues.

## Supporting Information

S1 Methods Methylation-specific PCR condition. Bisulfite-treated DNA was subjected to a MS-PCR using primer pairs designed to specifically amplify the target genes. The reaction solution (15 μL) contained HotStart Taq Premix (7.5 μL) (RBC Bioscience, Taipei, Taiwan), bisulfite-treated DNA (0.6 μL), and 0.6-μL aliquots of forward and reverse primers. The primer sequences for *p16*, *hMLH1*, and *MGMT* have been described previously.[14] The polymerase chain reaction (PCR) cycling conditions for the methylated and unmethylated primers involved denaturation at 95°C for 10 minutes, followed by 35 cycles at 95°C for 30 seconds, annealing at 62°C, 60°C, and 53°C for 35 seconds, and 72°C for 30 seconds, and a final extension at 72°C for 4 minutes. The PCR products were mixed with a DNA dye (Bioman, Taipei, Taiwan), subjected to horizontal gel electrophoresis on a 2% agarose gel for 25 minutes, and stained with ethidium bromide for 10 minutes. The results were analyzed under ultraviolet (UV) transillumination which was shown in S1 Fig. **Statistical methods.** In this study, patients were classified according to the methylation of 3 target genes. The primary outcome measures were CRC recurrence and mortality. The person-years of follow-up were used to calculate the CRC recurrence and mortality rates, starting on the date of surgical resection and continuing until the date of diagnosis of CRC recurrence, date of death, or December 31, 2012. A Kaplan-Meier analysis and a Cox proportional hazard model were used to analyze patient methylation status and clinical information at study entry, and their associations with CRC recurrence or mortality during follow-up, after adjusting for potential confounding covariates. The adjusted hazard ratios (HRs) and 95% confidence intervals (CIs) were calculated to evaluate methylation status as a predictor of risk of CRC recurrence. The potential confounding variables in the model were sex, age at surgery (treated as a continuous variable), tumor location, and adjuvant chemotherapy. The adjusted HRs were then calculated for each category. The associations between DNA methylation status and risk of CRC recurrence and mortality were further evaluated using analyses stratified according to the baseline clinical cancer stage (I–IV). To test a linear trend across the clinical cancer stages and methylation statuses in subtypes of tissues, the incidence rate of CRC recurrence in each category was used as a continuous variable in a multivariable model.
(DOCX)

S1 Fig Agarose gel was represented the *p16* gene promoter methylated or unmethylated status of CRC patients. T: tumor tissue; N: normal tissue; MR: marker for reference of PCR product size; U: unmethylation; M: methylation. For the quality of methylation-specific PCR, experimental controls were presented by WBC from healthy people, and two human colorectal adenocarcinoma cell lines (HT29 and DLD1). Water was for negative control for this experiment.
(TIF)

S1 Results Our results of the associations between the various cancer stages and CRC recurrence and mortality were observed a significant stepwise increase in CRC recurrence and mortality among the stage I–IV patients (*p* < 0.001). The stage III and IV patients were associated with significantly higher risk of CRC recurrence compared with the stage I patients, with adjusted HRs (95% CI) of 19.37 (2.59–145.05) and 56.36 (7.42–428.09), respectively. The stage IV patients were associated with significantly higher risk of mortality compared with the stage I patients, with an adjusted HR (95% CI) of 5.76 (1.99–16.61). We included the stage I and II patients in the local stage subgroup, and the stage III and IV patients in the advanced stage subgroup. The patients in the advanced stage subgroup were associated with higher risk of CRC recurrence and mortality compared with the patients in the local stage subgroup, with adjusted HRs (95% CI) of 6.73 (3.56–12.71) and 1.75 (0.94–3.28), respectively.
(DOCX)
