# Supplementary material for: DNA Methylation Combinations in Adjacent Normal Colon Tissue Predict Cancer Recurrence: Evidence from a Clinical Cohort Study
Source: PLoS One. 2015 Mar 27;10(3):e0123396. doi: 10.1371/journal.pone.0123396 (PMC4376718; doi:10.1371/journal.pone.0123396)
Supplement: S1 Methods — Bisulfite-treated DNA was subjected to a MS-PCR using primer pairs designed to specifically amplify the target genes. The reaction solution (15 μL) contained HotStart Taq Premix (7.5 μL) (RBC Bioscience, Taipei, Taiwan), bisulfite-treated DNA (0.6 μL), and 0.6-μL aliquots of forward and reverse primers. The primer sequences for p16, hMLH1, and MGMT have been described previously.[14] The polymerase chain reaction (PCR) cycling conditions for the methylated and unmethylated primers involved denaturation at 95°C for 10 minutes, followed by 35 cycles at 95°C for 30 seconds, annealing at 62°C, 60°C, and 53°C for 35 seconds, and 72°C for 30 seconds, and a final extension at 72°C for 4 minutes. The PCR products were mixed with a DNA dye (Bioman, Taipei, Taiwan), subjected to horizontal gel electrophoresis on a 2% agarose gel for 25 minutes, and stained with ethidium bromide for 10 minutes. The results were analyzed under ultraviolet (UV) transillumination which was shown in S1 Fig. Statistical methods. In this study, patients were classified according to the methylation of 3 target genes. The primary outcome measures were CRC recurrence and mortality. The person-years of follow-up were used to calculate the CRC recurrence and mortality rates, starting on the date of surgical resection and continuing until the date of diagnosis of CRC recurrence, date of death, or December 31, 2012. A Kaplan-Meier analysis and a Cox proportional hazard model were used to analyze patient methylation status and clinical information at study entry, and their associations with CRC recurrence or mortality during follow-up, after adjusting for potential confounding covariates. The adjusted hazard ratios (HRs) and 95% confidence intervals (CIs) were calculated to evaluate methylation status as a predictor of risk of CRC recurrence. The potential confounding variables in the model were sex, age at surgery (treated as a continuous variable), tumor location, and adjuvant chemotherapy. The adjusted H [file pone.0123396.s001.docx]

**S1 Methods**

*Methylation-specific PCR condition*

Bisulfite-treated DNA was subjected to a MS-PCR using primer pairs designed to specifically amplify the target genes. The reaction solution (15 μL) contained HotStart Taq Premix (7.5 μL) (RBC Bioscience, Taipei, Taiwan), bisulfite-treated DNA (0.6 μL), and 0.6-μL aliquots of forward and reverse primers. The primer sequences for *p16*, *hMLH1*, and *MGMT* have been described previously.[^14^](#_ENREF_14) The polymerase chain reaction (PCR) cycling conditions for the methylated and unmethylated primers involved denaturation at 95°C for 10 minutes, followed by 35 cycles at 95°C for 30 seconds, annealing at 62°C, 60°C, and 53°C for 35 seconds, and 72°C for 30 seconds, and a final extension at 72°C for 4 minutes. The PCR products were mixed with a DNA dye (Bioman, Taipei, Taiwan), subjected to horizontal gel electrophoresis on a 2% agarose gel for 25 minutes, and stained with ethidium bromide for 10 minutes. The results were analyzed under ultraviolet (UV) transillumination which was shown in Supplemental figure.

*Statistical methods*

In this study, patients were classified according to the methylation of 3 target genes. The primary outcome measures were CRC recurrence and mortality. The person-years of follow-up were used to calculate the CRC recurrence and mortality rates, starting on the date of surgical resection and continuing until the date of diagnosis of CRC recurrence, date of death, or December 31, 2012. A Kaplan-Meier analysis and a Cox proportional hazard model were used to analyze patient methylation status and clinical information at study entry, and their associations with CRC recurrence or mortality during follow-up, after adjusting for potential confounding covariates. The adjusted hazard ratios (HRs) and 95% confidence intervals (CIs) were calculated to evaluate methylation status as a predictor of risk of CRC recurrence. The potential confounding variables in the model were sex, age at surgery (treated as a continuous variable), tumor location, and adjuvant chemotherapy. The adjusted HRs were then calculated for each category. The associations between DNA methylation status and risk of CRC recurrence and mortality were further evaluated using analyses stratified according to the baseline clinical cancer stage (I–IV). To test a linear trend across the clinical cancer stages and methylation statuses in subtypes of tissues, the incidence rate of CRC recurrence in each category was used as a continuous variable in a multivariable model.
