# Supplementary figures and images for: DNA Methylation Combinations in Adjacent Normal Colon Tissue Predict Cancer Recurrence: Evidence from a Clinical Cohort Study
Source: PLoS One. 2015 Mar 27;10(3):e0123396. doi: 10.1371/journal.pone.0123396 (PMC4376718; doi:10.1371/journal.pone.0123396)

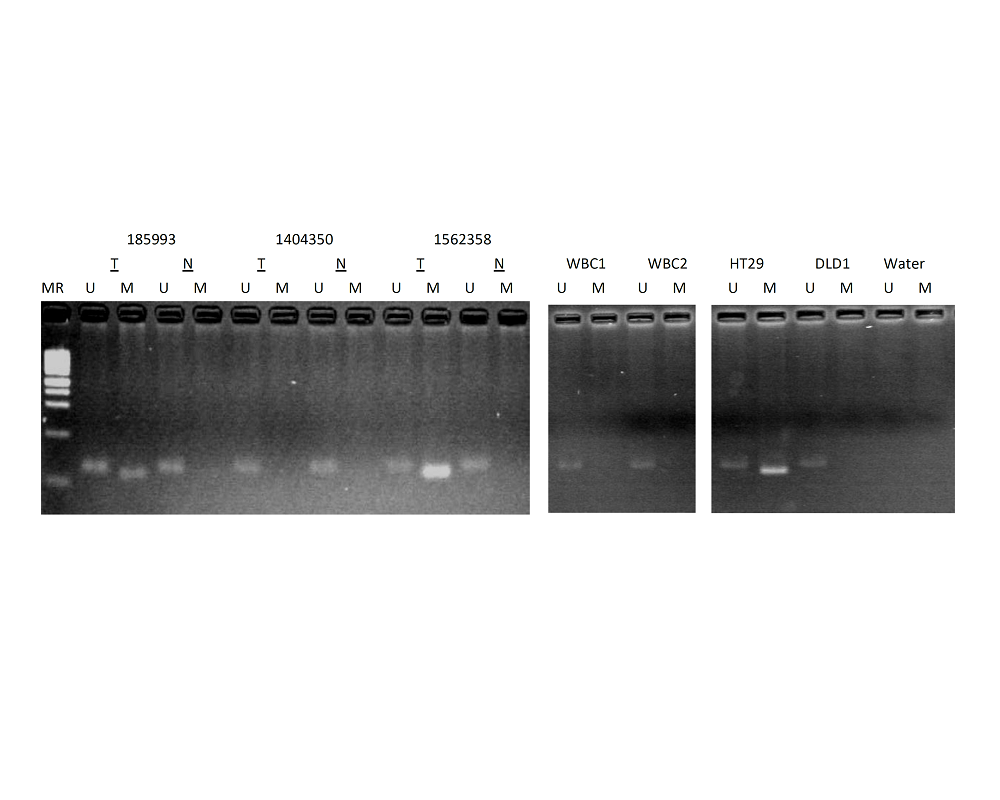

Supplement: S1 Fig — T: tumor tissue; N: normal tissue; MR: marker for reference of PCR product size; U: unmethylation; M: methylation. For the quality of methylation-specific PCR, experimental controls were presented by WBC from healthy people, and two human colorectal adenocarcinoma cell lines (HT29 and DLD1). Water was for negative control for this experiment. (TIF) [file pone.0123396.s002.tif]
