# Supplementary material for: DNA Methylation Combinations in Adjacent Normal Colon Tissue Predict Cancer Recurrence: Evidence from a Clinical Cohort Study
Source: PLoS One. 2015 Mar 27;10(3):e0123396. doi: 10.1371/journal.pone.0123396 (PMC4376718; doi:10.1371/journal.pone.0123396)
Supplement: S1 Results — The stage III and IV patients were associated with significantly higher risk of CRC recurrence compared with the stage I patients, with adjusted HRs (95% CI) of 19.37 (2.59–145.05) and 56.36 (7.42–428.09), respectively. The stage IV patients were associated with significantly higher risk of mortality compared with the stage I patients, with an adjusted HR (95% CI) of 5.76 (1.99–16.61). We included the stage I and II patients in the local stage subgroup, and the stage III and IV patients in the advanced stage subgroup. The patients in the advanced stage subgroup were associated with higher risk of CRC recurrence and mortality compared with the patients in the local stage subgroup, with adjusted HRs (95% CI) of 6.73 (3.56–12.71) and 1.75 (0.94–3.28), respectively. (DOCX) [file pone.0123396.s003.docx]

**S1 Results**

Our results of the associations between the various cancer stages and CRC recurrence and mortality were observed a significant stepwise increase in CRC recurrence and mortality among the stage I–IV patients (*p* < 0.001). The stage III and IV patients were associated with significantly higher risk of CRC recurrence compared with the stage I patients, with adjusted HRs (95% CI) of 19.37 (2.59–145.05) and 56.36 (7.42–428.09), respectively. The stage IV patients were associated with significantly higher risk of mortality compared with the stage I patients, with an adjusted HR (95% CI) of 5.76 (1.99–16.61). We included the stage I and II patients in the local stage subgroup, and the stage III and IV patients in the advanced stage subgroup. The patients in the advanced stage subgroup were associated with higher risk of CRC recurrence and mortality compared with the patients in the local stage subgroup, with adjusted HRs (95% CI) of 6.73 (3.56–12.71) and 1.75 (0.94–3.28), respectively.
